# Predicting rapid radiographic progression in difficult-to-treat rheumatoid arthritis: insights from long-term follow-up

**DOI:** 10.1093/rheumatology/keaf515

**Published:** 2025-10-01

**Authors:** Kerem Abacar, Andrea Di Matteo, Paula David, Shouvik Dass, Paul Emery, Kulveer Mankia, Benazir Saleem, Dennis McGonagle

**Affiliations:** Leeds Institute of Rheumatic and Musculoskeletal Medicine, University of Leeds, Chapel Allerton Hospital, Leeds, UK; Leeds Institute of Rheumatic and Musculoskeletal Medicine, University of Leeds, Chapel Allerton Hospital, Leeds, UK; NIHR Leeds Biomedical Research Centre, Leeds Teaching Hospitals NHS Trust, Leeds, UK; Leeds Institute of Rheumatic and Musculoskeletal Medicine, University of Leeds, Chapel Allerton Hospital, Leeds, UK; Department of Internal Medicine Sheba Medical Center- Tel Hashomer, Ramat Gan, Israel; NIHR Leeds Biomedical Research Centre, Leeds Teaching Hospitals NHS Trust, Leeds, UK; Leeds Institute of Rheumatic and Musculoskeletal Medicine, University of Leeds, Chapel Allerton Hospital, Leeds, UK; NIHR Leeds Biomedical Research Centre, Leeds Teaching Hospitals NHS Trust, Leeds, UK; Leeds Institute of Rheumatic and Musculoskeletal Medicine, University of Leeds, Chapel Allerton Hospital, Leeds, UK; NIHR Leeds Biomedical Research Centre, Leeds Teaching Hospitals NHS Trust, Leeds, UK; NIHR Leeds Biomedical Research Centre, Leeds Teaching Hospitals NHS Trust, Leeds, UK; Leeds Institute of Rheumatic and Musculoskeletal Medicine, University of Leeds, Chapel Allerton Hospital, Leeds, UK; NIHR Leeds Biomedical Research Centre, Leeds Teaching Hospitals NHS Trust, Leeds, UK

**Keywords:** difficult-to-treat rheumatoid arthritis, rapid radiographic progression, poly-refractory rheumatoid arthritis, persistent inflammatory refractory and non-inflammatory refractory rheumatoid arthritis

## Abstract

**Objectives:**

To investigate the long-term trajectory of radiographic progression in difficult-to-treat rheumatoid arthritis (D2T RA) and poly-refractory RA (pr-RA) patients and to evaluate the impact of ultrasound-based persistent inflammatory refractory RA (PIRRA) and non-inflammatory refractory RA (NIRRA) classification on predicting rapid radiographic progression (RRP, ≥5 mSvdH units/year).

**Methods:**

Radiographic damage was assessed using the modified Sharp/van der Heijde (mSvdH) score in EULAR-defined D2T RA patients. PIRRA and NIRRA subgroups were classified based on a single ultrasound time point assessing grayscale and power Doppler synovitis. The impact of time-integrated CRP and swollen joint counts (SJC) on radiographic progression was examined.

**Results:**

Among 254 D2T RA patients, 114 had serial radiographs with a mean follow-up of 9 years. The mean annual mSvdH progression was 2.8 units with both time-integrated CRP (*P < *0.001) and the PIRRA patients (*n* = 43) having significantly greater annual radiographic progression (3.3 units in PIRRA *vs* 2.4 units in NIRRA, *P = *0.025). In multivariable analysis, older age (*P = *0.017) and swollen joint count (*P = *0.009) were independently associated with RRP. Additionally, RRP was observed in 50% of pr-RA patients (*n* = 14) *vs* 19.4% in other D2T RA cases (*P = *0.048).

**Conclusion:**

Although pr-RA cases are an uncommon subgroup, half of them demonstrated RRP, emphasizing the need for more aggressive treatment approaches. In contrast, many D2T RA patients exhibited comparatively slow radiographic progression indicating that many D2T RA cases are at least partially treated. These findings underscore the heterogeneity within D2T RA and highlight the need for additional strategies for the pr-RA subgroup.

Rheumatology key messages

- In difficult-to-treat RA, swollen joint counts and CRP predict rapid radiographic progression.
- Polyrefractory RA is rare but clinically important, with 50% showing rapid radiographic progression.
- Ultrasound distinguishes inflammatory versus non-inflammatory refractory RA, guiding prognosis and treatment decisions.

## Introduction

Biologic and targeted synthetic disease-modifying anti-rheumatic drugs (b/tsDMARDs) have transformed the management of rheumatoid arthritis (RA) [1, 2]. Despite the availability of various therapeutic modalities, including T cell modulation, B cell depletion therapies and numerous cytokine-targeted agents, a subset of RA patients remains treatment refractory [3]. The concept of treatment resistance in RA covers a broad spectrum of clinical scenarios [4, 5]. To address this complexity, the European League Against Rheumatism (EULAR) introduced the term ‘difficult-to-treat rheumatoid arthritis’ (D2T RA), providing a comprehensive definition that captures the spectrum of treatment resistance [6].

The prevalence of D2T RA ranges between 5% and 20% with disparate patient groups encompassed within the definition [4, 5, 7, 8]. Within this spectrum, we identified a subset of patients classified as poly-refractory RA (pr-RA), namely those that have failed all available treatment classes [9]. The prevalence of pr-RA patients among the RA population receiving b/tsDMARD treatment was 2.7% in our institution [9]. Assessment tools such as the Disease Activity Score-28 (DAS-28), commonly used to evaluate disease activity and treatment resistance, capture both inflammatory and non-inflammatory mechanisms of disease. Notably, non-inflammatory pain mechanisms contribute to treatment resistance in patients unresponsive to intensive anti-inflammatory therapies [5]. Based on these distinctions, we have suggested that D2T RA can be stratified into two classes: persistent inflammatory refractory RA (PIRRA) and non-inflammatory refractory RA (NIRRA) [5]. Among D2T RA patients, including those with pr-RA, ∼40% lacked objective signs of joint inflammation on ultrasound (US), categorizing them as NIRRA, while the remaining cases were classified as PIRRA based on the presence of US-detected inflammation at a recent single time point in disease activity assessment [4].

A critical prognostic indicator in RA is joint damage progression, as the severity of radiographic damage is associated with functional disability [10]. Accordingly, the EULAR task force has suggested that, in addition to non-responsiveness to two or more classes of b/tsDMARDs, a rapid radiographic progression exceeding 5 units per year can be used to identify patients with D2T RA [11–14]. Prior to the biologic era the mean annualized radiographic erosion progression in RA patients was 8.6 Sharp units [15]. However, radiographic progression rates in real world D2T RA is not documented and its correlation with persistent inflammation remains unclear. In this work, we investigated the trajectory of long-term radiographic progression in D2T RA and pr-RA patients and evaluated the impact of PIRRA and NIRRA status on rapid radiographic progression (RRP).

## Methods

### Study population

This study was conducted as a retrospective observational cohort analysis. Patients were identified from the biologics clinics at Leeds Teaching Hospitals NHS Trust and were included if they were diagnosed with RA between 1996 and 2025, fulfilled the EULAR definition of D2T RA, and had at least two conventional radiographs (hands and/or feet) taken at different time points. Clinical and laboratory data were extracted retrospectively from routine care records, prioritizing the time point closest to the baseline radiograph. Those meeting the EULAR criteria for D2T RA were included if they met the following conditions: in addition to failing ≥2 b/tsDMARDs after unsuccessful treatment with conventional synthetic DMARDs (csDMARDs), patients were required to exhibit at least one indicator of active or progressive disease. These indicators included moderate or higher disease activity (DAS28-ESR >3.2 or Clinical Disease Activity Index (CDAI) >10), signs and/or symptoms suggestive of active disease such as extra-articular manifestations, inability to taper glucocorticoid therapy, RRP or a significant reduction in quality of life [6]. As routine radiographs are not part of the systematic follow-up evaluation at our institution, RRP was not used to select D2T RA.

Data collected included demographic characteristics (age, gender, disease duration, smoking status, etc.), serological status such as rheumatoid factor (RF), anti-citrullinated protein antibody (ACPA), comorbidities such as osteoporosis [16], as well as all b/tsDMARD treatments previously administered and the patients’ responses to these therapies, assessed using the DAS28 for RA-CRP (DAS28-CRP). CRP levels taken at the time radiographs were acquired were recorded to assess their association with radiographic progression.

Within the D2T RA cohort, patients who failed (due to either inefficacy or intolerance) ≥1 medication from each available class of b/tsDMARDs—including anti-TNF agents (infliximab, adalimumab, etanercept, certolizumab, golimumab), anti-IL-6 agents (tocilizumab, sarilumab), anti-CD20 agents (rituximab), T cell co-stimulation modulators (abatacept), and Janus kinase (JAK) inhibitors (tofacitinib, baricitinib, filgotinib, upadacitinib)—were classified as having pr-RA [9]. However, in seronegative patients, where rituximab is typically not utilized in our centre, failure of the anti-TNF, anti-IL-6, abatacept and JAK inhibitor classes was defined as ‘seronegative pr-RA’.

### Ultrasonographic definition of PIRRA and NIRRA

Ultrasound data were used only if the scan had been performed after patients had fulfilled the EULAR criteria for D2T RA. Scans were requested when there was clinical suspicion of active synovitis, and only patients with at least one clinically swollen joint were referred for musculoskeletal US. The US scan focused on the clinically swollen joints. US scans were performed by rheumatologists experienced in the use of US using a GE Logiq E9 machine (GE Healthcare, Chicago, IL, USA) with a linear ML 15–6 MHz transducer. The pulse refresh frequency was set to 700–1000 Hz and the Doppler frequency was set to 10 MHz. The sonographers were blinded to patient data but were aware of physical examination findings (i.e. swelling). As previously reported, only joints deemed to be clinically swollen were scanned by a sonographer since joint swelling is a strong predictor of damage [9]. On the other hand, clinically tender but non swollen joints were not scanned. Synovitis on US was defined as a combination of greyscale changes and power Doppler signal (greyscale ≥1 + power Doppler ≥1) as defined by EULAR/Outcome Measures in Rheumatology [17]. The presence/absence of synovitis on US was used to define the PIRRA and NIRRA phenotype, respectively [9].

### Radiographic evaluation

All available hand and foot radiographs of D2T RA patients were reviewed. Radiographs taken at two different time points were scored using the modified Sharp/van der Heijde (mSvdH) scoring method by two independent rheumatology experts (K.A. and A.D.M.), blinded to each other’s assessments and all patient data. In patients with multiple radiographs, the earliest and latest radiographs were evaluated to detect changes over the longest follow-up period. Of the patients assessed, 96 met the criteria for D2T RA on their second radiograph but not the first, three patients met the criteria on both radiographs, and 15 patients did not meet the criteria on either radiograph. Inter-observer concordance (ICC) analysis was conducted to evaluate consistency. The calculated ICC values for the first- and second-hand radiographs between both readers were 0.954 and 0.976, respectively, while the calculated ICC values for the first- and second-foot radiographs were 0.928 and 0.927. The progression in radiographic scores between the two sets of images was calculated, and the ‘annual radiographic progression rate’ was determined by dividing the total mSvdH score progression by the time interval between the two radiographs. Additionally, in line with the D2T RA criteria and as previously defined, patients with an annual mSvdH score increase of 5 units or more were classified as having RRP [6, 18].

### Statistics

The data were analysed using the SPSS Statistics v 22.0 (IBM Corp., Armonk, NY, USA). Continuous variables were expressed as mean (standard deviation) for normally distributed data and as median (interquartile range, IQR) for non-normally distributed data. Categorical variables were described using number and percentage.

For patients whose annual CRP levels could be tracked, the ‘time-integrated CRP’ values were calculated using the area-under-the-curve (AUC) method [19]. To facilitate comparison with the annual rate of radiographic progression, the annual time-integrated CRP rate was also computed.

Univariable logistic regression models were used to assess the association between clinical, laboratory and ultrasound parameters and the presence of rapid radiographic progression (RRP), defined as a binary outcome. Variables that were significant at *P < *0.05 in the univariable analysis were subsequently included in the multivariable logistic regression model. All statistical tests were two-sided, and a significance level of *P < *0.05 was considered for all analyses.

Formal ethical approval was not required as the work was a retrospective analysis, conducted under the framework of an approved service evaluation (audit) of the Leeds Teaching Hospitals Trust’s specialist RA Biologics Clinic as previously described. The study reporting adhered to the Strengthening the Reporting of Observational Studies in Epidemiology (STROBE) guidelines.

## Results

Among the 254 D2T RA patients identified from a total cohort of 1600 RA patients who were prescribed b/tsDMARD therapy, 114 patients [mean (s.d.) age 60.9 (12.8) years; 81% female; 79% seropositive) had radiographs available at two time points (Supplementary Fig. 1). The mean (s.d.) disease duration at the last visit was 15.9 (4.5) years, while the mean (s.d.) disease duration at the time of the first X-ray was 4.56 (3.7) years. Fourteen (12.3%) patients were pr-RA. Hand radiographs at two time points were available for all patients (*n* = 114), including those with US assessments (*n* = 74); however, as foot radiographs were not performed in patients without foot pain, the mSvdH score could only be calculated for the 80 patients with available foot radiographs, of whom 51 had US assessments. The mean (s.d.) interval between baseline and final radiographs was 9 (4.1) years. In the 74 patients who underwent power Doppler US, the PIRRA/NIRRA ratio was 43/31 (58%/42%). Details regarding patient demographics and disease-related variables can be found in Table 1.

**Table 1.** Demographic data and disease-related characteristics of patients

	All patients (*n* = 114)
Age, mean (s.d.), years	60.9 (12.8)
Age at diagnosis, mean (s.d.), years	44.7 (12.1)
Gender (F/M ratio)	92/22
Disease duration, mean (s.d.), years	15.9 (4.5)
Body mass index, mean (s.d.), kg/m^2^	29.5 (7.3)
Smoker ever, n (%)	11 (9.6)
Number of bDMARD usage, mean (s.d.)	4.1 (1.6)
Number of bDMARD usage from different classes, mean (s.d.)	3.7 (1)
pr-RA, n (%)	14 (12.3)
TJC at last visit, mean (s.d.)	11.8 (6.8)
SJC at last visit, mean (s.d.)	5 (4.8)
PIRRA/NIRRA rate, n (%)^a^	43 (58.1)/31 (41.9)
DAS28 at last visit, mean (s.d.)	5.2 (1)
RF positivity, n (%)	81 (71.1)
ACPA positivity, n (%)	84 (73.7)
CRP (mg/l) level at last visit, mean (s.d.)	16.8 (28.1)

ACPA: anti-citrullinated peptide antibody; bDMARD: biologic DMARD; DAS28: Disease Activity Score–28 joints; F: female; M: male; NIRRA: non-inflammatory refractory RA; PIRRA: persistent inflammatory refractory RA; pr-RA: polyrefractory RA; RF: rheumatoid factor; SJC: swollen joint count; TJC: tender joint count. ^a^Percentages calculated among 74 patients who underwent ultrasound assessment.

**Figure 1.**
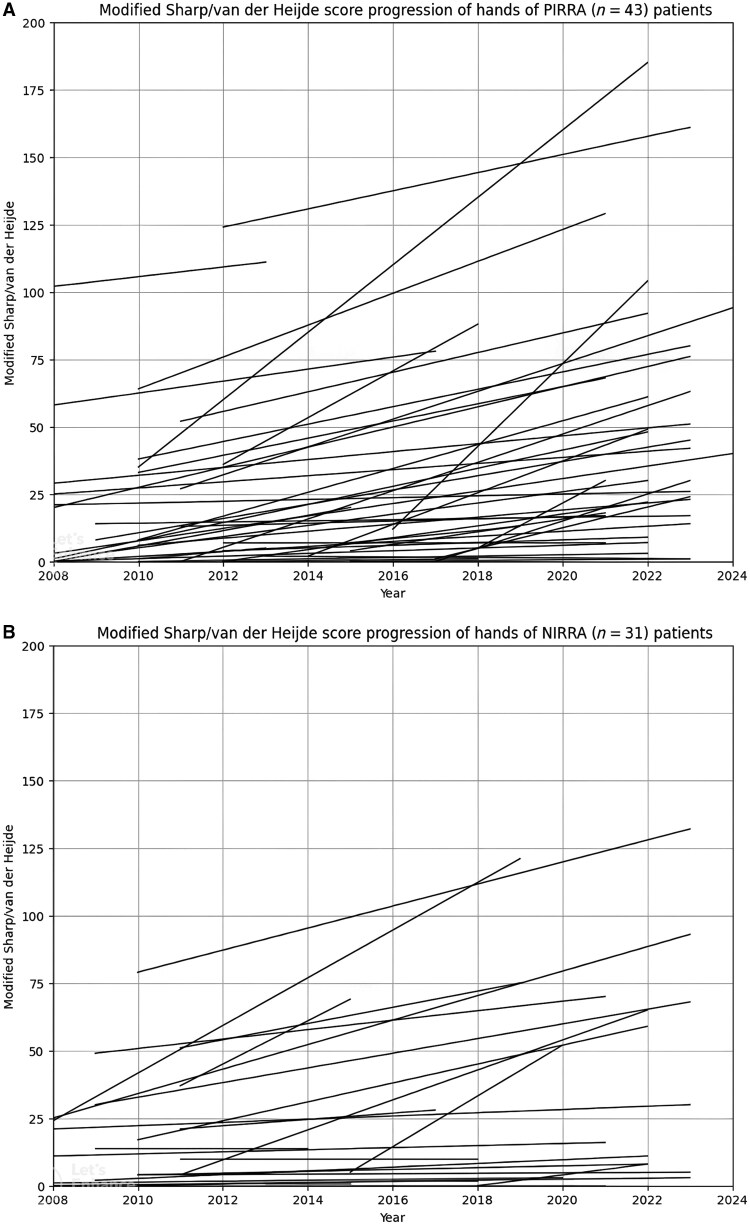
Individual progression process of PIRRA and NIRRA patients in X-ray images according to modified Sharp/van der Heijde score. NIRRA: non-inflammatory refractory RA; PIRRA: persistent inflammatory refractory RA

The mean (s.d.) mSvdH hand score of all patients on the initial and final radiographs was 14.9 (23.7) and 36.5 (40.5), respectively (Fig. 1). The corresponding mean (s.d.) feet scores were 6.7 (14.2) and 13.7 (17.7), while the total mSvdH scores were 22.5 (36.6) and 48.7 (52.2), respectively. The mean (s.d.) annual progression of the mSvdH score was 2.8 (2.8), with mean (s.d.) rates of 2.3 (2.7) for the hands and 0.8 (1.1) for the feet (Table 2). Age (*P < *0.001), age at diagnosis (*P = *0.006), disease duration (*P = *0.005) and the number of prior bDMARDs used (*P = *0.033) were significantly positively correlated with annual hand mSvdH score progression. Additionally, both age (*P = *0.027) and disease duration (*P = *0.038) demonstrated significant correlations with annual total mSvdH score progression. The annual progression of the hand mSvdH score was significantly higher in men compared with women (*P = *0.04) and in patients with osteoporosis compared with those without (*P = *0.024). However, no statistically significant relationship was found between smoking status and radiographic progression rates (Table 3).

**Table 2.** Comparison of patients’ modified Sharp/van der Heijde scores according to PIRRA/NIRRA and pr-RA/non pr-RA classifications

	All patients (*n* = 114)	PIRRA (*n* = 43)	NIRRA (*n* = 31)	P-value	pr-RA (*n* = 14)	non-pr-RA (*n* = 100)	P-value
Time from first to last X-ray, mean (s.d.), years	9 (4.1)	10.1 (3.8)	9.1 (4.3)	0.257	10.6 (4.2)	8.8 (4.1)	0.122
mSvdH in hands at first X-ray, mean (s.d.)	14.9 (23.7)	17.8 (27.2)	13.4 (19)	0.591	21.1 (19)	14.1 (24)	0.057
mSvdH in hands at last X-ray, mean (s.d.)	36.5 (40.5)	46.1 (44.6)	30.6 (38.6)	**0.041**	54.8 (35.7)	34 (40.6)	**0.014**
mSvdH in feet at first X-ray, mean (s.d.)	6.7 (14.2)	8.9 (19)	5.1 (8.1)	0.562	9.3 (19.9)	6.3 (13.2)	0.943
mSvdH in feet at last X-ray, mean (s.d.)	13.7 (17.7)	17.7 (21.4)	12.5 (14.8)	0.212	16.1 (23.8)	13.3 (16.8)	0.664
Hand mSvdH progression, mean (s.d.)	21.6 (26.7)	28.3 (29.6)	17.2 (25.3)	**0.022**	33.6 (23.1)	19.9 (26.9)	**0.007**
Feet mSvdH progression, mean (s.d.)	6.8 (9.5)	8.9 (11)	7.4 (10.7)	0.362	6.8 (7.1)	6.8 (9.8)	0.421
Yearly mSvdH hand progression, mean (s.d.)	2.3 (2.7)	3 (3.2)	1.8 (2.6)	**0.025**	3.8 (2.8)	2 (2.7)	**0.011**
Yearly mSvdH feet progression, mean (s.d.)	0.8 (1.1)	1 (1.3)	0.9 (1.3)	0.367	0.7 (0.8)	0.8 (1.2)	0.688
Total mSvdH at first X-ray, mean (s.d.)	22.5 (36.6)	27 (47.2)	22.5 (26.2)	0.865	33.1 (37.5)	20.8 (36.5)	0.122
Total mSvdH at last X-ray, mean (s.d.)	48.7 (52.2)	59.6 (57.3)	44.5 (53.5)	0.103	75 (58.1)	45 (50.6)	0.052
Total mSvdH progression, mean (s.d.)	26.2 (29)	33.1 (26.4)	22 (31.1)	**0.037**	43.2 (28.7)	23.7 (28.3)	**0.026**
Yearly total mSvdH progression, mean (s.d.)	2.8 (2.8)	3.3 (2.5)	2.4 (3.2)	0.056	4.1 (3.1)	2.6 (2.7)	0.089
Yearly time-integrated CRP level, mean (s.d.)	12.9 (12)	15.8 (13.2)	11.2 (7.9)	0.17	19.7 (8.6)	11.9 (12.2)	**<0.001**

Bold values indicate statistically significant *P*-values (*P < *0.05). mSvdH: modified Sharp/van der Heijde score; NIRRA: non-inflammatory refractory RA; PIRRA: persistent inflammatory refractory RA; pr-RA: polyrefractory RA.

**Table 3.** Correlations between disease features and yearly radiographic progression of the patients according to modified Sharp/van der Heijde score

	Yearly modified Sharp/van der Heijde score progression					
	Hand		Feet		Total	
Correlations	*r*	*P*	*r*	*P*	*r*	*P*
Age	**0.31**	**<0.001**	0.09	0.38	**0.25**	**0.027**
Age at diagnosis	**0.26**	**0.006**	−0.07	0.53	0.18	0.13
Disease duration	**0.27**	**0.005**	0.19	0.11	**0.242**	**0.038**
Number of bDMARD usage	**0.2**	**0.033**	0.07	0.55	0.18	0.12
Number of bDMARD usage from different classes	0.17	0.08	0.06	0.59	0.17	0.15
TJC at last visit	−0.08	0.41	0.17	0.14	0.06	0.64
SJC at last visit	0.17	0.08	0.07	0.56	0.2	0.07
VAS at last visit	0.04	0.66	**0.24**	**0.04**	0.17	0.14
DAS28 at last visit	0.03	0.73	0.21	0.07	0.18	0.11
CRP level at baseline	0.15	0.14	0.08	0.51	0.14	0.22
Yearly time-integrated CRP level	**0.5**	**<0.001**	**0.3**	**0.01**	**0.48**	**<0.001**

**Comparisons**	**Rate**	***P***	**Rate**	***P***	**Rate**	***P***

Female, mean (s.d.)	2.07 (2.7)	**0.04**	0.74 (1.1)	0.228	2.64 (2.9)	0.207
Male, mean (s.d.)	3.21 (3)		1.03 (1.1)		3.55 (2.7)	
Seropositive, mean (s.d.)	2.32 (2.7)	0.435	0.89 (1.2)	**0.031**	2.87 (2.7)	0.178
Seronegative, mean (s.d.)	2.18 (3)		0.3 (0.6)		2.58 (3.7)	
Smoking ever, mean (s.d.)	2.71 (2.8)	0.912	2.58 (2.5)	0.714	0.19 (0.4)	0.137
Absence of smoking history, mean (s.d.)	2.83 (2.8)		2.26 (2.8)		0.85 (1.1)	
Presence of osteoporosis, mean (s.d.)	3.32 (2.8)	**0.024**	0.87 (1.3)	0.662	3.96 (3.4)	0.161
Absence of osteoporosis, mean (s.d.)	2.1 (2.7)		0.77 (1.1)		2.57 (2.6)	

Bold values indicate statistically significant *P*-values (*P < *0.05). bDMARD: biologic DMARD; DAS28: Disease Activity Score–28 joints; SJC: swollen joint count; TJC: tender joint count.

### Polyrefractory RA and rapid radiographic progression

Based on the final radiographs, the annual progression of mSvdH hand scores was also significantly greater in the pr-RA group than non pr-RA (yearly mean (s.d.) mSvdH scoring progression of pr-RA *vs* non pr-RA patients, respectively: 3.8 (2.8) *vs* 2 (2.7) unit, *P = *0.011) (Table 2). The time-integrated CRP levels were significantly elevated in pr-RA patients (*P < *0.001). In this work, although the use of RRP for D2T RA patient selection was not used, 18/80 patients had RRP and these were older (*P = *0.032), were more likely pr-RA (*P = *0.043) and had a higher number of swollen joint counts (SJC; *P = *0.004) at last visit in the univariable binary logistic regression analysis. The RRP rate was 50% in the pr-RA group and 19.4% in those without (*P = *0.048). In the multivariable logistic regression analysis created with these three parameters, statistically significant relationships were found with age (*P = *0.017) and SJC at last visit parameters (*P = *0.009) (Table 4).

**Table 4.** Univariate and multivariate logistic regression analysis for rapid radiographic progression (RRP)

	Univariate		Multivariate	
	*P*	OR (95% CI)	*P*	OR (95% CI)
Age	**0.032**	1.056 (1.005, 1.109)	**0.017**	1.070 (1.012, 1.131)
Age at diagnosis	0.097	1.044 (0.992, 1.1)		
Disease duration	0.153	1.095 (0.967, 1.241)		
Female gender	0.738	0.802 (0.221, 2.914)		
BMI	0.421	0.997 (0.99, 1.004)		
pr-RA	**0.043**	4.154 (1.045, 16.504)	0.206	2.796 (0.569, 13.754)
SJC	**0.004**	1.206 (1.062, 1.370)	**0.009**	1.219 (1.051, 1.414)
Osteoporosis	0.235	2.137 (0.611, 7.473)		
PIRRA	0.472	1.636 (0.427, 6.264)		
Seropositivity	0.492	0.630 (0.169, 2.355)		
Yearly time-integrated CRP levels	0.06	1.052 (0.998, 1.11)		

Multicollinearity was evaluated by calculating variance inflation factor (VIF) and tolerance values. All VIF values were below 5, and tolerance values exceeded 0.2, confirming the absence of significant multicollinearity. Rapid radiographic progression (RRP) defined as >5 mSvdH units/per year. Bold values indicate statistically significant *P*-values (*P < *0.05). OR: odds ratio; PIRRA: persistent inflammatory refractory RA; pr-RA: polyrefractory RA; SJC: swollen joint count.

### Persistent inflammatory refractory RA and non-inflammatory refractory RA

**Figure 2.**
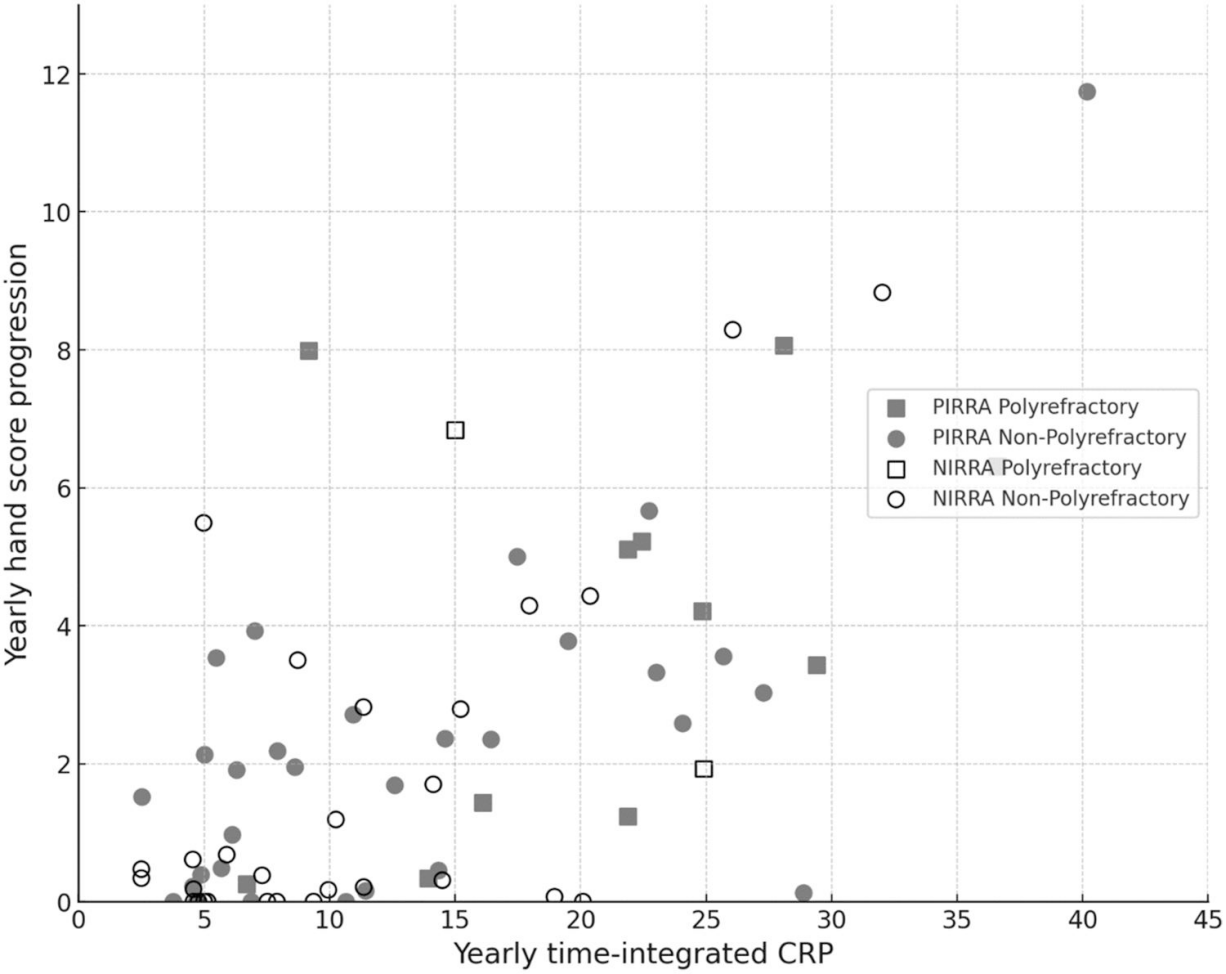
Demonstration of correlation between yearly time-integrated CRP and yearly hand score progression on a patient basis according to PIRRA/NIRRA and poly-refractory classification. NIRRA: non-inflammatory refractory RA; PIRRA: persistent inflammatory refractory RA

PIRRA patients (*n* = 43) who had a single US between 2021 and 2024 exhibited significantly higher baseline mSvdH hand erosion and joint space narrowing scores compared with NIRRA patients (*n* = 31) [mean (s.d.) for erosion: 14.9 (20) *vs* 12.1 (19.5), *P = *0.049; mean (s.d.) for joint space narrowing: 31.2 (27) *vs* 18.5 (22.2), *P = *0.026 for PIRRA and NIRRA, respectively]. The total progression up to the final radiographs, as well as the annual mSvdH hand score progression, was also significantly greater in PIRRA patients than in NIRRA patients [PIRRA mean (s.d.): 3.0 (3.2), NIRRA mean (s.d.): 1.8 (2.6); *P = *0.025] (Table 2). In addition, the annual mSvdH total score progression was higher in PIRRA patients than in NIRRA patients. However, this difference did not reach statistical significance (*P = *0.056), likely reflecting a recent single time point for US that did not capture prior active D2T disease and the relatively small number of patients (Table 2). Figure 1 illustrates the individual progression of mSvdH hand scores in PIRRA and NIRRA patients. Additionally, the proportion of PIRRA was significantly higher among the 14 pr-RA patients (PIRRA/NIRRA ratio: 12/2, *P = *0.02) (Fig. 2). (Baseline differences between the PIRRA/NIRRA and pr-RA/non-pr-RA groups are summarized in Supplementary Table 1.)

### Time integrated CRP

Yearly time-integrated CRP levels were statistically significantly correlated with the annual mSvdH score progression in hands (*r* = 0.5, *P < *0.001), feet (*r* = 0.3, *P = *0.01) and total (*r* = 0.48, *P < *0.001) (Table 2). In addition, yearly time-integrated CRP levels in pr-RA patients were statistically significantly higher than those without pr-RA (Table 3). Figure 2 illustrates the correlation between yearly time-integrated CRP levels and annual mSvdH hand score progression, stratified by PIRRA/NIRRA and pr-RA/non-pr-RA subgroups. When the impact of the yearly time-integrated CRP was excluded from the analysis, a statistically significant correlation was observed between SJC and yearly mSvdH hand and total score progression (*r* = 0.403, *P = *0.006 for total score, and *r* = 0.382, *P = *0.01 for hand score).

## Discussion

In this work we show that factors known to predict radiographic progression for RA also apply to D2T RA, with SJC, annual time-integrated CRP, age and pr-RA status being the strongest predictors. Although radiographic progression in D2T RA was lower than in historical placebo cohorts, the RRP phenotype was common in the pr-RA subgroup. D2T RA is a challenging subset within the broader RA population, characterized by refractoriness to standard therapies, high disease burden and frequent co-morbidities, leading to significant disability and diminished quality of life [20–22]. Radiographic joint damage progression is strongly linked to disability and a diminished quality of life [23–25]. In this regard, we investigated hand and foot radiographic progression in a D2T RA population over a long follow-up period. Overall, we found that radiographic progression in the D2T RA group fell below the threshold of the defined RRP value (defined as 5 units per year) but in the pr-RA group half of the cases presented an ongoing and sustained RRP picture. This emphasizes the need for therapy refinement in this uncommon subgroup.

As historically shown in the ATTRACT trial, biologic therapies are pivotal in reducing radiographic progression. The annual radiographic progression (mSvdH score) was 6 units in placebo patients with an ACR20 response, compared with 0.1 in the infliximab group [12]. Compared with that yardstick clinical trial, our D2T RA group had lower radiographic progression with an annualized radiographic progression rate of 2.8 units, which was substantially lower than that observed in the placebo arm of the ATTRACT trial, suggesting a degree of therapeutic benefit and reduced structural damage progression in cases failing two therapy classes post-csDMARDs. Importantly, prior studies have demonstrated that even clinical non-responders to bDMARDs experience reduced radiographic progression, suggesting that the structural protective effects of these therapies may be independent of clinical response [26]. This aligns with our findings, as radiographic progression in the D2T RA group remained low despite ongoing disease activity, underscoring the dissociation between clinical response and structural protection in D2T RA. Overall, radiographic progression in the D2T RA group, as expected, aligned with joint swelling, CRP and positive ultrasound scan for PDUS change, indicating the parameters that can identify cases at higher risk of progression. These findings are in line with established risk models for radiographic progression in RA [18, 27–31]. In the model proposed by Vastesaeger *et al.*, SJC28, RF, CRP and ESR were identified as significant predictors of RRP, based on data from the ATTRACT and ASPIRE trials [12, 18, 32]. Similarly, in a TNFi trial-based matrix model including 1371 RA patients, SJC28, CRP and physician global assessment were significant predictors of radiographic progression [31]. Consistent with these findings, our study also demonstrated that SJC and CRP remain key determinants of radiographic worsening in D2T RA.

However, in the small pr-RA subgroup, RRP was evident in half of the cases [9, 33]. Acute phase reactant elevation such as CRP and the presence of US detected synovitis were, as expected, linked to damage progression. Although there was no significant difference in tender joint counts and VAS between the PIRRA and NIRRA groups as previously reported [9], the PIRRA group with objective evidence of synovitis had significantly higher annual radiographic progression (3.3 *vs* 2.4 respectively for PIRRA *vs* NIRRA group) even though ultrasound was performed at one recent time point only. Although numbers are small, these findings suggest that even if symptoms do not differ, structural disease progression related to objective inflammation at a single time point is linked to RA progression. This highlights the complementary role of ultrasound alongside routinely used clinical markers in refining risk models and guiding therapeutic decision-making, particularly for high-risk subgroups like pr-RA patients. Supporting this, findings from the BeST study demonstrated that elevated APR and baseline erosions were strong predictors of RRP in patients receiving methotrexate monotherapy, with this risk being substantially reduced by TNFi or corticosteroid therapy [34]. Notably, while seropositivity was associated with RRP in that study, our findings indicate that its predictive value is more limited in D2T RA, where inflammation markers such as CRP play a more dominant role in driving structural progression. These insights reinforce the importance of objective inflammatory assessment in guiding treatment strategies, particularly in the pr-RA subset.

Our study has certain limitations. Among the D2T RA patient cohort, radiographic imaging at two distinct time points was available in less than half of the total population (114 out of 254 patients), reflecting real-world practice rather than a structured imaging protocol. However, patients included in this analysis did not substantially differ from the broader D2T RA cohort previously described at our centre, supporting the generalizability of the imaging-based findings. While some patients were classified as having D2T RA prior to their initial radiograph and others after their first or second X-ray, the extended duration of follow-up and the generally slow rate of structural progression in RA mitigate the impact of this temporal variation. We acknowledge that assessing radiographic progression using only the earliest and latest available radiographs may not fully reflect potential non-linear trajectories of joint damage. Nonetheless, this method offers a pragmatic and consistent approach for evaluating long-term damage in routine care, where imaging is not performed at standardized intervals. Additionally, cross-sectional ultrasound was only performed in joints with clinical swelling, which may introduce a degree of selection bias. However, this aligns with the intent to define phenotypes under symptomatic conditions. As the PIRRA and NIRRA subtypes were designed to reflect distinct mechanisms of treatment resistance in patients with active disease, we believe that this strategy enhances the clinical relevance of the phenotyping framework.

In conclusion, while many D2T RA patients exhibit slow radiographic progression, persistent inflammatory markers—including time-integrated CRP, PIRRA on ultrasound and SJC—were strongly associated with accelerated structural damage. Notably, pr-RA patients exhibited a significantly higher rate of RRP, affecting 50% of cases, emphasizing the need for more targeted therapeutic approaches. These findings suggest that D2T RA is a heterogeneous entity, encompassing subsets with varying degrees of treatment resistance, where persistent inflammation remains a key driver of progression. Integrating ultrasound-based classification with traditional clinical markers may enhance risk stratification and facilitate more personalized treatment strategies in refractory RA.

## Supplementary Material

keaf515_Supplementary_Data

## Data Availability

Data are available from the corresponding author upon reasonable request.

## References

[1] Smolen JS , Landewé RBM, Bergstra SA et al EULAR recommendations for the management of rheumatoid arthritis with synthetic and biological disease-modifying antirheumatic drugs: 2022 update. Ann Rheum Dis 2023;82:3–18. 36357155 10.1136/ard-2022-223356

[2] Aletaha D , Smolen JS. Diagnosis and management of rheumatoid arthritis: a review. JAMA 2018;320:1360–72. 30285183 10.1001/jama.2018.13103

[3] Tan Y , Buch MH. 'Difficult to treat’ rheumatoid arthritis: current position and considerations for next steps. RMD Open 2022;8:e002387. 10.1136/rmdopen-2022-002387 PMC9335059 35896282

[4] Buch MH. Defining refractory rheumatoid arthritis. Ann Rheum Dis 2018;77:966–9. 29588276 10.1136/annrheumdis-2017-212862

[5] Buch MH , Eyre S, McGonagle D. Persistent inflammatory and non-inflammatory mechanisms in refractory rheumatoid arthritis. Nat Rev Rheumatol 2021;17:17–33. 33293696 10.1038/s41584-020-00541-7

[6] Nagy G , Roodenrijs NM, Welsing PM et al EULAR definition of difficult-to-treat rheumatoid arthritis. Ann Rheum Dis 2021;80:31–5. 33004335 10.1136/annrheumdis-2020-217344 PMC7788062

[7] Kearsley-Fleet L , Davies R, De Cock D; BSRBR-RA Contributors Group et al Biologic refractory disease in rheumatoid arthritis: results from the British Society for Rheumatology Biologics Register for Rheumatoid Arthritis. Ann Rheum Dis 2018;77:1405–12. 29980575 10.1136/annrheumdis-2018-213378 PMC6161665

[8] de Hair MJH , Jacobs JWG, Schoneveld JLM, van Laar JM. Difficult-to-treat rheumatoid arthritis: an area of unmet clinical need. Rheumatology (Oxford) 2018;57:1135–44. 29029308 10.1093/rheumatology/kex349

[9] David P , Di Matteo A, Hen O et al Poly-refractory rheumatoid arthritis: an uncommon subset of difficult to treat disease with distinct ınflammatory and noninflammatory phenotypes. Arthritis Rheumatol 2024;76:510–21. 38059326 10.1002/art.42767

[10] van der Heijde D. Radiographic progression in rheumatoid arthritis: does it reflect outcome? Does it reflect treatment? Ann Rheum Dis 2001;60 Suppl 3:iii47–50. 11890653 10.1136/ard.60.90003.iii47 PMC1766669

[11] Emery P , Fleischmann R, van der Heijde D et al The effects of golimumab on radiographic progression in rheumatoid arthritis: results of randomized controlled studies of golimumab before methotrexate therapy and golimumab after methotrexate therapy. Arthritis Rheum 2011;63:1200–10. 21305524 10.1002/art.30263

[12] Smolen JS , Han C, Bala M; ATTRACT Study Group et al Evidence of radiographic benefit of treatment with infliximab plus methotrexate in rheumatoid arthritis patients who had no clinical improvement: a detailed subanalysis of data from the anti-tumor necrosis factor trial in rheumatoid arthritis with concomitant therapy study. Arthritis Rheum 2005;52:1020–30. 15818697 10.1002/art.20982

[13] Takeuchi T , Yamanaka H, Ishiguro N et al Adalimumab, a human anti-TNF monoclonal antibody, outcome study for the prevention of joint damage in Japanese patients with early rheumatoid arthritis: the HOPEFUL 1 study. Ann Rheum Dis 2014;73:536–43. 23316080 10.1136/annrheumdis-2012-202433 PMC4151516

[14] Kremer JM , Blanco R, Brzosko M et al Tocilizumab inhibits structural joint damage in rheumatoid arthritis patients with inadequate responses to methotrexate: results from the double-blind treatment phase of a randomized placebo-controlled trial of tocilizumab safety and prevention of structural joint damage at one year. Arthritis Rheum 2011;63:609–21. 21360490 10.1002/art.30158

[15] Hulsmans HM , Jacobs JW, van der Heijde DM et al The course of radiologic damage during the first six years of rheumatoid arthritis. Arthritis Rheum 2000;43:1927–40. 11014342 10.1002/1529-0131(200009)43:9<1927::AID-ANR3>3.0.CO;2-B

[16] Risk WSGoAoF, Osteoporosis iAtSfP. Assessment of fracture risk and its application to screening for postmenopausal osteoporosis: report of a WHO study group. Geneva: World Health Organization, 1994. 7941614

[17] D'Agostino M-A , Terslev L, Aegerter P et al Scoring ultrasound synovitis in rheumatoid arthritis: a EULAR-OMERACT ultrasound taskforce-Part 1: definition and development of a standardised, consensus-based scoring system. RMD Open 2017;3:e000428. 28948983 10.1136/rmdopen-2016-000428 PMC5597799

[18] Vastesaeger N , Xu S, Aletaha D, St Clair EW, Smolen JS. A pilot risk model for the prediction of rapid radiographic progression in rheumatoid arthritis. Rheumatology (Oxford) 2009;48:1114–21. 19589891 10.1093/rheumatology/kep155

[19] Plant MJ , Williams AL, O'Sullivan MM et al Relationship between time-integrated C-reactive protein levels and radiologic progression in patients with rheumatoid arthritis. Arthritis Rheum 2000;43:1473–7. 10902748 10.1002/1529-0131(200007)43:7<1473::AID-ANR9>3.0.CO;2-N

[20] Dey M , Nagy G, Nikiphorou E. Comorbidities and extra-articular manifestations in difficult-to-treat rheumatoid arthritis: different sides of the same coin? Rheumatology (Oxford) 2023;62:1773–9. 36205537 10.1093/rheumatology/keac584

[21] Roodenrijs NMT , van der Goes MC, Welsing PMJ et al Difficult-to-treat rheumatoid arthritis: contributing factors and burden of disease. Rheumatology (Oxford) 2021;60:3778–88. 33331946 10.1093/rheumatology/keaa860

[22] Watanabe R , Okano T, Gon T et al Difficult-to-treat rheumatoid arthritis: current concept and unsolved problems. Front Med (Lausanne) 2022;9:1049875. 36353219 10.3389/fmed.2022.1049875 PMC9637686

[23] Scott DL , Pugner K, Kaarela K et al The links between joint damage and disability in rheumatoid arthritis. Rheumatology (Oxford) 2000;39:122–32. 10725061 10.1093/rheumatology/39.2.122

[24] Scott DL , Symmons DP, Coulton BL, Popert AJ. Long-term outcome of treating rheumatoid arthritis: results after 20 years. Lancet 1987;1:1108–11. 2883443 10.1016/s0140-6736(87)91672-2

[25] Welsing PM , van Gestel AM, Swinkels HL, Kiemeney LA, van Riel PL. The relationship between disease activity, joint destruction, and functional capacity over the course of rheumatoid arthritis. Arthritis Rheum 2001;44:2009–17. 11592361 10.1002/1529-0131(200109)44:9<2009::AID-ART349>3.0.CO;2-L

[26] Emery P , Genovese MC, van Vollenhoven R et al Less radiographic progression with adalimumab plus methotrexate versus methotrexate monotherapy across the spectrum of clinical response in early rheumatoid arthritis. J Rheumatol 2009;36:1429–41. 19369462 10.3899/jrheum.081018

[27] Vanier A , Smolen JS, Allaart CF et al An updated matrix to predict rapid radiographic progression of early rheumatoid arthritis patients: pooled analyses from several databases. Rheumatology (Oxford) 2020;59:1842–52. 31722413 10.1093/rheumatology/kez542

[28] Fautrel B , Granger B, Combe B et al Matrix to predict rapid radiographic progression of early rheumatoid arthritis patients from the community treated with methotrexate or leflunomide: results from the ESPOIR cohort. Arthritis Res Ther 2012;14:R249. 23164197 10.1186/ar4092 PMC3674616

[29] Curtis JR , Brahe CH, Østergaard M et al Predicting risk for radiographic damage in rheumatoid arthritis: comparative analysis of the multi-biomarker disease activity score and conventional measures of disease activity in multiple studies. Curr Med Res Opin 2019;35:1483–93. 30777458 10.1080/03007995.2019.1585064

[30] Lillegraven S , Paynter N, Prince FHM et al Performance of matrix-based risk models for rapid radiographic progression in a cohort of patients with established rheumatoid arthritis. Arthritis Care Res (Hoboken) 2013;65:526–33. 23044765 10.1002/acr.21870 PMC3594116

[31] Smolen JS , Kang YM, Yoo W-H et al Radiographic progression based on baseline characteristics from TNF inhibitor biosimilar studies in patients with rheumatoid arthritis. Arthritis Res Ther 2020;22:188. 32795341 10.1186/s13075-020-02267-z PMC7427775

[32] Smolen JS , Van Der Heijde DMFM, St Clair EW; Active-Controlled Study of Patients Receiving Infliximab for the Treatment of Rheumatoid Arthritis of Early Onset (ASPIRE) Study Group et al Predictors of joint damage in patients with early rheumatoid arthritis treated with high-dose methotrexate with or without concomitant infliximab: results from the ASPIRE trial. Arthritis Rheum 2006;54:702–10. 16508926 10.1002/art.21678

[33] Lidar M , Rimar D, David P et al CD-19 CAR-T cells for polyrefractory rheumatoid arthritis. Ann Rheum Dis 2025;84:370–2. 39919909 10.1136/ard-2024-226437

[34] Goekoop-Ruiterman YPM , de Vries-Bouwstra JK, Allaart CF et al Clinical and radiographic outcomes of four different treatment strategies in patients with early rheumatoid arthritis (the BeSt study): a randomized, controlled trial. Arthritis Rheum 2005;52:3381–90. 16258899 10.1002/art.21405

