# Supplementary material for: Predicting rapid radiographic progression in difficult-to-treat rheumatoid arthritis: insights from long-term follow-up
Source: Rheumatology (Oxford). 2025 Oct 1;65(1):keaf515. doi: 10.1093/rheumatology/keaf515 (PMC12862374; doi:10.1093/rheumatology/keaf515)
Supplement: keaf515_Supplementary_Data [file keaf515_supplementary_data.docx]

**Supplementary Figure S1:** Flow chart of D2T RA patients

RA patients followed with b/tsDMARDs treatment (n=1600)

Patients who underwent X-ray at two different timepoints (n=114)

Patients with only hand X-rays

(n=114)

PIRRA (n=43)

pr-RA (n=12)

non pr-RA (n=31)

pr-RA (n=2)

non pr-RA (n=29)

D2T RA patients

(n=254)

Both hands and feet (n=80)

Non-RRP (n=62)

RRP (n=18)

NIRRA (n=31)

RA: Rheumatoid arthritis, b/tsDMARDs: Biologic/targeted synthetic disease-modifying anti-rheumatic drugs, D2T RA: Difficult-to-Treat Rheumatoid Arthritis, PIRRA: Persistent Inflammatory Refractory RA, NIRRA: Non-Inflammatory Refractory RA, RRP: Rapid radiographic progression, pr-RA: Polyrefractory rheumatoid arthritis.

**Alt text:** Flow chart detailing the selection and classification of D2T RA patients from an initial b/tsDMARD-treated cohort (n=1600) to subgroups based on radiographic availability and PIRRA/NIRRA status.

**Supplementary Table S1.** Baseline Clinical and Laboratory Characteristics Across PIRRA/NIRRA and Poly-refractory Subgroups in D2T RA Patients with Serial Radiographs

| **Variable** | **PIRRA** | **NIRRA** | **p (PIRRA vs NIRRA)** | **Poly-refractory** | **Non-Poly-refractory** | **p (pr-RA vs non-prRA)** |
| --- | --- | --- | --- | --- | --- | --- |
| Age, mean (SD) | 55.2 (12.3) | 54.8 (11.7) | 0.812 | 56.1 (13.0) | 54.5 (12.1) | 0.456 |
| SJC, mean (SD) | 7.0 (3.2) | 1.0 (1.1) | **<0.001** | 6.5 (3.0) | 2.5 (2.2) | **0.002** |
| TJC, mean (SD) | 5.5 (2.8) | 4.8 (2.5) | 0.215 | 5.2 (2.6) | 4.9 (2.4) | 0.612 |
| Yearly time integrated CRP, mean (SD) | 15.2 (5.6) | 5.0 (2.1) | **<0.001** | 14.0 (5.2) | 6.0 (2.5) | **0.001** |
| RF positive, n (%) | 18 (78.3%) | 14 (70.0%) | 0.542 | 16 (76.2%) | 16 (66.7%) | 0.488 |
| CCP positive, n (%) | 17 (73.9%) | 13 (65.0%) | 0.567 | 15 (71.4%) | 15 (62.5%) | 0.512 |
| Female, n (%) | 12 (52.2%) | 10 (50.0%) | 0.891 | 11 (52.4%) | 12 (50.0%) | 0.882 |
| Osteoporosis, n (%) | 5 (21.7%) | 4 (20.0%) | 0.912 | 5 (23.8%) | 4 (16.7%) | 0.642 |
| Disease duration at first X-ray, mean (SD) | 8.0 (3.5) | 7.5 (3.2) | 0.612 | 8.2 (3.6) | 7.8 (3.3) | 0.701 |

(PIRRA: Progression-Integrated Radiographic Refractory Arthritis, NIRRA: Non-Integrated Radiographic Refractory Arthritis, pr-RA: Polyrefractory Rheumatoid Arthritis, SJC: Swollen Joint Count, TJC: Tender Joint Count, CRP: C-reactive protein, RF: Rheumatoid Factor, CCP: Anti-Cyclic Citrullinated Peptide, SD: Standard Deviation, D2T RA: Difficult-to-Treat Rheumatoid Arthritis, BMI: Body Mass Index).
